# An evaluation of organ dose modulation on a GE optima CT660‐computed tomography scanner

**DOI:** 10.1120/jacmp.v17i3.5724

**Published:** 2016-05-08

**Authors:** Matthew T. Dixon, Robert J. Loader, Gregory C. Stevens, Nick P. Rowles

**Affiliations:** ^1^ Department of Clinical and Radiation Physics Plymouth Hospitals NHS Trust Plymouth UK

**Keywords:** Monte Carlo simulations, phantom study, computed tomography, tube current modulation, organ dose modulation

## Abstract

Organ Dose Modulation or ODM (GE Healthcare, Milwaukee, WI) was evaluated to characterize changes in ${\text{CTDI}}_{\text{vol}}$, image noise, effective dose, and organ dose saving to patients. Three separate investigations were completed: a tube current modulation phantom was scanned with and without ODM, a ${\text{CTDI}}_{\text{vol}}$ phantom was scanned with ODM, and Monte Carlo simulations were performed. ODM was found to reduce the ${\text{CTDI}}_{\text{vol}}$ by approximately 20% whilst increasing the noise by approximately 14%. This was reflected in the dose distribution, where the anterior peripheral dose was reduced by approximately 40% whilst the identical posterior dose remained largely unaffected. Enabling ODM for the entire scan would reduce the effective dose by approximately 24%; however, this saving reduces to 5% if the images are matched for ${\text{CTDI}}_{\text{vol}}$. These savings mostly originated from reductions in dose to the stomach, breasts, colon, bladder, and liver. ODM has the effect of a global reduction in ${\text{CTDI}}_{\text{vol}}$ with an associated increase in image noise. The benefit of ODM was found to be reduced when the dose‐saving contribution from the reduced ${\text{CTDI}}_{\text{vol}}$ was removed. Given that there is a higher contribution to effective dose throughout the body from the anterior projections, consideration should be given to applying ODM throughout.

PACS number(s): 87.10.Rt, 87.53.Bn, 87.57.C‐, 87.57.Q‐

## I. INTRODUCTION

The use of computed tomography (CT) has risen dramatically in recent years; for example, in England between 2000 and 2010 the use of CT almost tripled.^(1)^ This has been accompanied by an increased focus on the associated radiation dose, particularly in the United States with the Image Gently^(2)^ and Image Wisely campaigns.^(3)^ In the UK, the Ionising Radiation (Medical Exposure) Regulations mandate optimization, paying special attention to higher dose modalities (Regulation 7, Part 7).^(4)^ Many applications are being developed and introduced by manufacturers to reduce the radiation burden to populations exposed to CT.

One particular concern is the relatively high absorbed dose to radiosensitive organs, such as breasts and eyes. Attempts have been made to reduce this by direct shielding of organs in the primary beam. However, concerns have been raised regarding the detrimental effect on image quality. For example, Servaes and Zhu,^(5)^ Huggett et al.,^(6)^ and Foely et al.^(7)^ advise against using breast shields due to the negative effect on image quality, noting that dose reduction would be better achieved through reducing the overall tube current.

A contemporary solution offered by some CT manufacturers is to reduce the dose to radiosensitive anterior organs by reducing the tube current when the X‐ray tube is in the anterior position. Two examples of the implementation of this are X‐CARE (Siemens HealthCare, Erlangen, Germany)^(8)^ and Organ Dose Modulation (GE Healthcare, Milwaukee, WI).^(9)^ The authors are not aware of any other known implementations of this particular technique at the time of writing. X‐CARE and ODM operate on the same principle, with two main differences. Firstly, X‐CARE increases the tube current when outside the anterior projections whereas ODM does not, and secondly, each selects different angles to apply the modulation. ODM uses a 180° arc for body protocols and a 90° arc for head protocols,^(10)^ whereas X‐CARE uses a 120° arc exclusively.^(11)^ A 120° arc has been shown to be insufficient to entirely encompass the breasts^(12)^ and a 180° arc may, therefore, be advantageous.

Many studies have considered organ‐based tube current modulation, both in comparison to breast shielding and general scanning. Matsubara et al.^(13)^ observe that breast dose can be reduced by 20% by using such a technique with a negligible effect on image noise or CT numbers, contrary to the effect on CT numbers and noise when using copper shielding. Hoang et al. ^(11)^ observe that the dose to the thyroid and lens can be reduced, again with minimal effect on image quality. However, both these studies utilize X‐CARE for the comparison. To the best of our knowledge there are few published studies on implementation of ODM.

The objectives of this study are therefore twofold: to ensure that image quality is not compromised by applying ODM, and to evaluate the efficacy of ODM as a dose reduction mechanism in terms of stochastic risk to the patient.

## II. METHODS

### A. Image quality investigation

All images were acquired using an Optima CT660 CT scanner (GE Healthcare, Milwaukee, WI). All scans used the following parameters: 120 kVp, 40 mm beam collimation, 5 mm image thickness, 500 mm reconstruction field of view, 500 mA (max), 10 mA (min), 1 s rotation time, AutomA enabled, and SmartmA enabled. AutomA and SmartmA are GE implementations of z‐axis and xy‐axis tube current modulation (TCM), respectively. They attempt to achieve a specified, constant level of image noise independent of patient morphology specified by a ‘Noise Index’ (higher Noise Index means lower tube current). These techniques also incorporate a minimum and maximum tube current (mA) preventing the current from going beyond chosen extremes.

To investigate the effect of ODM on image quality, a CelT phantom (Design Reality, Denbighshire, UK) was used. The CelT phantom is a phantom with five different thickness segments, designed to assess the system's automatic exposure control (AEC) function in CT. It contains four material inserts throughout its length: polymethyl methacrylate (PMMA), polyethylene, polyoxymethylene, water, as well as air. The CelT phantom was scanned in its entirety with three separate Noise Indexes (10, 20, and 40) in axial mode and once in helical mode (with a Noise Index of 20) both with ODM on and off. Given subsequently observed variation in noise and dose distribution behavior for matched Noise Index scans, these scans were then repeated, instead matching the volumetric‐computed tomography dose index (${\text{CTDI}}_{\text{vol}}$) of each methodology (ODM off and on) by altering the Noise Index accordingly. This was considered a more equivalent parameter to match as Noise Index is a proprietary definition by GE, whereas ${\text{CTDI}}_{\text{vol}}$ is an established technique. In addition, early results indicated that noise values were comparable when ${\text{CTDI}}_{\text{vol}}$ rather than Noise Index were matched. The exact values used are shown in Table 1.

**Figure 1:**
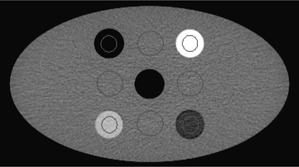
Positioning of regions of interest within CelT phantom shown on the smallest section.

2.5 cm and 1.5 cm (diameter) regions of interest were drawn in axially identical positions throughout the CelT phantom in the center of each material insert, as well as in four PMMA regions spaced evenly between the inserts, as shown in Fig. 1. For each region of interest the average Hounsfield number and standard deviation (SD) were recorded.

**Table 1:** ${\text{CTDI}}_{\text{vol}}$ and Noise Index settings for the scans performed.

*Organ Dose Modulation*	*Scan Mode*	*Scanner Predicted* ${\text{CTDI}}_{\text{vol}}$ *(mGy)*	*Noise Index*
*Matched Noise Index*			
Off	Axial	8.18	10
Off	Axial	2.08	20
Off	Axial	0.91	40
Off	Helical	2.16	20
On	Axial	6.56	10
On	Axial	1.73	20
On	Axial	0.89	40
On	Helical	1.63	20
*Matched* ${\text{CTDI}}_{\text{vol}}$ *(matched to prior values of opposite ODM setting)*			
Off	Axial	6.57	11.15
Off	Axial	1.73	22.23
Off	Axial	0.89	42
Off	Helical	1.63	23.15
On	Axial	8.19	8.96
On	Axial	2.08	18
On	Axial	0.91	37
On	Helical	2.16	17.25

### B. Dose distribution investigation

ODM aims to reduce the anterior tube current by 40%.^(10)^ To investigate the success of this, a ${\text{CTDI}}_{\text{vol}}$ phantom was used. This phantom is a PMMA cylinder, 32 cm in diameter, with eight 1.5 cm holes equally spaced at 15 cm axially from the center, eight 1.5 cm diameter holes equally spaced at 7 cm axially from the center, and a single 1.5 cm diameter hole in the center. The scan was performed using identical parameters for the Noise Index of 20, axial scan in the previous section. Measurements were taken in each of the eight outer holes in the ${\text{CTDI}}_{\text{vol}}$ phantom using two 100 cm ionization chambers (Accu‐Pro 10X6‐3CT, Radcal Corporation, Monrovia, CA) to maximize time available on the CT scanner. The results from this test were also used to ensure that the scanner‐calculated ${\text{CTDI}}_{\text{vol}}$ was accurate by calculating the ${\text{CTDI}}_{\text{vol}}$ as described in the American Association of Physics in Medicine (AAPM) Report 96.^(14)^

### C. Effective dose investigation

Given that the purpose of ODM is to redistribute the dose to minimize the risk of harm to the patient, it was considered appropriate to quantify the benefit of redistributing the dose to the posterior of the patient. Given that the most likely adverse event (caused by radiation) expected from a CT scan would be the stochastic induction of a cancer, the effective dose (as defined by the International Commission on Radiological Protection)^(15)^ quantifies the overall stochastic detriment from a radiation exposure and was used.

To examine the relative contributions to effective dose, the Monte Carlo simulation program PCXMC^(16)^ was used. The simulations were based on the Optima CT660 CT scanner^(10)^ for different combinations of TCM and ODM. The system has an anode angle of 7° and a kVp of 120 was used exclusively. The focus to isocenter distance is 541 mm and the beam full width at half maximum (FWHM) in the Z direction is approximately 23 mm for a nominal 20 mm collimation.

PCXMC is limited in that only two filter materials could be specified, which was insufficient to completely model the filtration of the CT scanner in question. The technical reference manual provides a “quality equivalent filtration” (QEF), referring to a thickness of aluminium that would provide a similar spectrum. The actual filters and their quality equivalents are shown in Table 2. To clarify the similarity, we used the Monte Carlo software EGNRC (National Research Council Canada, Ottawa, ON, Canada) to compare the actual spectrum and the equivalent aluminium spectrum. The photon flux was found to agree within 5% overall and within 10% for the tungsten K‐edges.

**Figure 2:**
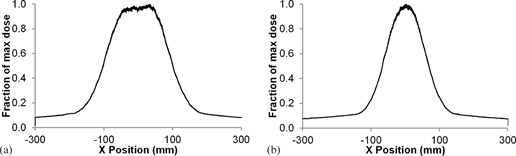
Bowtie filter profiles for (a) the large filter (left), and (b) small filter (right) in the x‐direction.

To better characterize the beam, a solid‐state dose sensor (Accu‐Pro DDX6‐W, Radcal Corporation, Monrovia, CA) was placed at the isocenter on top of a thin cardboard box (to minimize the contribution from scattered radiation). It was then irradiated with both bowtie filters using a stationary X‐ray tube to give an indication of the dose distribution from each filter. The sensor was then moved across the center of the scanner to obtain profiles in both the xy‐ and z‐planes. The measured profiles in the axial direction are shown in Fig. 2.

Given the small variation of the dose profile in the Z direction (<10%) and that the primary goal is to determine dose distributions in the xy‐plane, it was decided that a constant dose in the Z direction with a beam width of 23 mm could be assumed, reflecting the FWHM of the profile.

**Figure 3:**
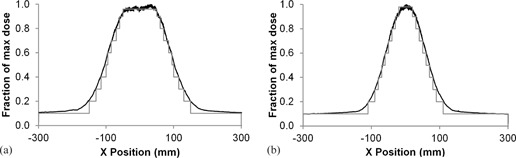
Inverse‐square corrected bowtie filter profiles for (a) the large filter (left) and (b) small filter (right) along with the approximated profile created in PCXMC, shown in gray.

PCXMC does not allow direct modification of the beam as it assumes a uniform field. To account for this, both bowtie fields were recreated using a combination of 10 fields in the x‐plane, each contributing 10% of the maximum dose recorded. The exceptions were the smallest fields, which contribute 8% and 6% for the small and large filters, respectively, to better represent the peak of the distribution. These fields can be seen in gray in Fig. 3. The measured profile was inverse‐square–corrected for each point to be perpendicular to the tube. The reason for this is that PCXMC models photons emanating from a point source and scales dose by the dose on the central axis of the beam. Therefore, the measured values must be corrected to consider appropriate relative contributions of points outside the central axis. This profile is shown in black in Fig. 3. The z‐plane was considered sufficiently uniform, as described earlier, and therefore it was not subdivided.

**Table 2:** A table showing the different filters within the Optima 660, shown in order of their position in the tube.

*Bowtie Filter*	*Aluminum (mm)*	*Graphite (carbon) (mm)*	*Aluminum (mm)*	*Copper (mm)*
Small (5.9 mm Al Quality Equivalent Filtration)	5.500	1.998	0.250	0.000
Large (8.6 mm Al Quality Equivalent Filtration)	5.500	1.998	0.250	0.075

Each of the 10 beams was simulated with a Z width of 23 mm (at the isocenter) at 10° intervals around the phantom. A PCXMC standard hermaphroditic patient was used (178.6 cm height, 73.2 kg mass) with arms absent from the phantom. The reference point was considered central to the geometric center of the patient. The simulations were performed from a Z position of 0 cm (the base of the torso) to a Z position of 70 cm (the top of the torso). Given that this investigation simply seeks to consider the relative contribution from different current distributions, exact measurement of dose was not required.

**Figure 4:**
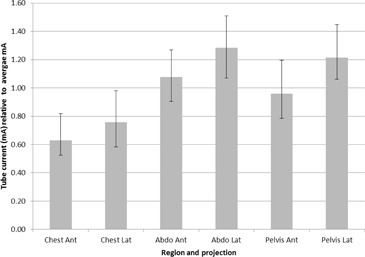
Median and interquartile ranges for tube currents in real patients. Each measurement was normalized by dividing by the patient's average mA.

In normal practice, the tube current is modulated in both the Z direction and XY directions to account for the changing patient shape. This function is performed automatically by AECs. For GE systems the Z direction modulation is termed AutomA and the XY direction modulation is termed SmartmA. These systems select the tube current for the primary scan based on the scan projection radiograph (scout) image. Again for GE systems, this modulation is divided into four quadrants, with anterior and posterior quadrants receiving a set dose in each rotation, and left and right quadrants similarly receiving a set dose in each rotation. To quantify the typical variation locally, a set of 20 chest‐abdomen‐pelvis patients were taken and the current for each quadrant and slice position recorded. Each value was assigned to “chest,” “abdomen,” or “pelvis” by anatomical location (chest considered to end at the base of the lungs, abdomen considered to end at the top of the iliac crest) and normalized by dividing by the average mA for the patient. The median normalized values across all patients for each category were used to scale the doses recorded from each of the simulated projections, as shown in Fig. 4.

## III. RESULTS

### A. Image quality investigation

Table 3 shows the resultant ${\text{CTDI}}_{\text{vol}}$ values. Given that the minimum mA was reached when using a Noise Index of 40, these results were excluded from the analysis. The reduction in ${\text{CTDI}}_{\text{vol}}$ when enabling ODM was approximately 20% (±5%). This is intuitively reasonable, based on the 40% reduction in mA for half the scan, which would average out to a net 20% reduction.

For each region of interest in each segment of the phantom for each scan the average Hounsfield number and standard deviation (SD) were found. The equivalent segments and scans were then compared between the scans with ODM enabled and disabled. Hounsfield number was compared absolutely, whilst standard deviation (noise) was compared relatively.

With matched ${\text{CTDI}}_{\text{vol}}$, there was an average of 0.13 (range: −3.63–4.20) absolute difference in Hounsfield number between the ODM enabled and disabled scans. The standard deviation was on average 1.6% (±0.6% two standard errors of the mean) higher with ODM enabled.

With matched Noise Indexes, there was an average of 0.76 (range: −2.67–4.07) absolute difference in Hounsfield number between the ODM enabled and disabled scans. The standard deviation was on average 14.3% (±2.2% 2 SD of the mean) higher with ODM enabled.

**Table 3:** ${\text{CTDI}}_{\text{vol}}$ values indicated by the scanner across different noise indexes, both with and without ODM.

*Noise Index*	*Set* ${\text{CTDI}}_{\text{vol}}$ *(ODM off)*	*Set* ${\text{CTDI}}_{\text{vol}}$ *(ODM on)*	*Ratio*
20 (Helical Mode)	2.16	1.63	0.75
10	8.18	6.56	0.80
20	2.08	1.73	0.83

### B. Dose distribution investigation

**Figure 5:**
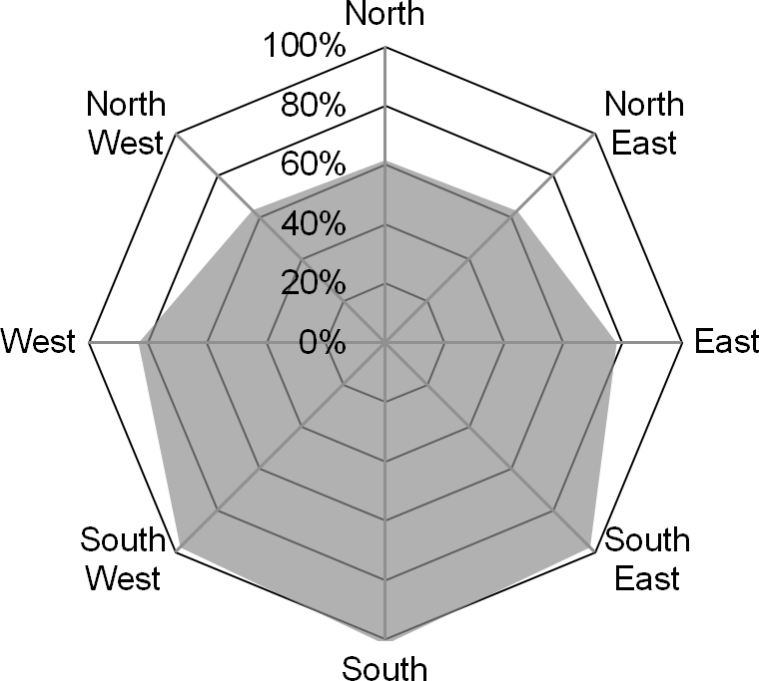
Dose with ODM enabled as a percentage of dose without ODM enabled.

The measured peripheral absorbed dose distribution in the PMMA ${\text{CTDI}}_{\text{vol}}$ phantom is shown in Fig. 5. As would be expected, the doses to anterior positions were reduced by approximately 40% with ODM enabled, corresponding to a 40% reduction in tube current over the anterior of the phantom. Left and right projections were reduced by approximately 20% and the posterior positions were almost completely unaffected. The scanner calculated ${\text{CTDI}}_{\text{vol}}$ was found to be accurate within approximately 5%.

### C. Effective dose investigation

Given that the results for the small and large bowtie filters were very similar and that the large bowtie filter would ordinarily be used for adult body patients, the large bowtie filter results only were included.

In the following results, values were normalized so that the total effective dose for exams with TCM disabled were the same as exams with TCM enabled. The reasoning behind this is that the effectiveness of TCM is well known and the focus of this paper is to consider the effectiveness of ODM with TCM off and on.

In addition, as it was observed (in the Materials & Methods section A) that the image noise increases in line with ${\text{CTDI}}_{\text{vol}}$ in the same way, whether ODM is enabled or not, and that matching ${\text{CTDI}}_{\text{vol}}$ values with ODM off or on results in matched noise values, the ODM doses were increased by a factor of 25% (1/0.8) to compensate for the 20% reduction in ${\text{CTDI}}_{\text{vol}}$ when enabling ODM. Therefore, anterior tube currents were 20% lower and posterior tube currents were 20% higher for the ODM analysis. To undo this correction, the ODM‐enabled results in 4, 7 can be multiplied by 0.8.

Organ dose values have been quoted in terms of their relative contribution to effective dose (the product of their equivalent dose and the tissue weighting factor) divided by the total effective dose for the equivalent exam with ODM disabled. This allowed a direct comparison of how utilizing ODM altered the total effective dose, as well as the dose to individual organs.

6, 7 show the change in effective dose when enabling ODM for different sections of the PCXMC phantom. It is seen that the effective dose was reduced at all points with this change.

4, 7 show the relative contribution to effective dose for the whole axial skeleton: chest, abdomen, and pelvis regions, respectively. Standard errors given by the PCXMC Monte Carlo simulation were combined in quadrature when summing the doses from the individual projections. These were all less than 0.05% for the individual organs and less than 0.2% for the effective dose.

**Table 4:** The total effective dose contribution from the given organ for the entire axial skeleton simulated for the large bowtie filter. This is relative to the total effective dose from a procedure where ODM is not enabled with identical ${\text{CTDI}}_{\text{vol}}$ and where TCM is set at the same setting. Only organs which contribute over 5% to the effective dose are shown. The values in brackets indicate the dose change from enabling ODM.

	*Normalized Relative Effective Dose Contribution*			
*Organ*	*ODM on TCM on*	*ODM off TCM on*	*ODM on TCM off*	*ODM off TCM off*
Effective dose (ICRP103) (mSv)	94.9% (−5.1%)	100.0%	94.8%	100.0%
Lungs (mGy)	12.3% (+0.1%)	12.2%	15.2% (+0.1%)	15.1%
Stomach (mGy)	14.8% (−2.1%)	16.9%	12.5% (−1.6%)	14.1%
Colon (mGy)	13.5% (−1.0%)	14.5%	11.7% (−0.8%)	12.5%
Breasts (mGy)	7.6% (−1.7%)	9.3%	10.1% (−2.2%)	12.3%
Active bone marrow (mGy)	11.9% (+1.0%)	10.9%	11.9% (+1.0%)	10.9%
Urinary bladder (mGy)	4.7% (−0.8%)	5.5%	4.3% (−0.7%)	5.0%
Liver (mGy)	5.0% (−0.3%)	5.3%	4.2% (−0.3%)	4.5%

**Table 5:** The total effective dose contribution from the given organ for the chest region simulated for the large bowtie filter. This is relative to the total effective dose from a procedure where ODM is not enabled with identical ${\text{CTDI}}_{\text{vol}}$ and where TCM is set at the same setting. Only organs which contribute over 5% to the effective dose are shown. The values in brackets indicate the dose change from enabling ODM.

	*Normalized Relative Effective Dose Contribution*			
*Organ*	*ODM on TCM on*	*ODM off TCM on*	*ODM on TCM off*	*ODM off TCM off*
Effective dose (ICRP103) (mSv)	94.7% (−5.3%)	100.0%	94.5% (+5.5%)	100.0%
Lungs (mGy)	33.1% (+0.3%)	32.8%	32.8% (+0.2%)	32.6%
Breasts (mGy)	22.4% (−5.0%)	27.4%	23.1% (−5.3%)	28.4%
Active bone marrow (mGy)	11.2% (+0.6%)	10.6%	11.2% (+0.6%)	10.6%
Oesophagus (mGy)	7.3% (+0.3%)	7.0%	7.3% (+0.3%)	7.0%
Thyroid (mGy)	5.0% (−0.5%)	5.5%	5.2% (−0.6%)	5.8%

**Table 6:** The total effective dose contribution from the given organ for the abdomen region simulated for the large bowtie filter. This is relative to the total effective dose from a procedure where ODM is not enabled with identical ${\text{CTDI}}_{\text{vol}}$ and where TCM is set at the same setting. Only organs which contribute over 5% to the effective dose are shown. The values in brackets indicate the dose change from enabling ODM.

	*Normalized Relative Effective Dose Contribution*			
*Organ*	*ODM on TCM on*	*ODM off TCM on*	*ODM on TCM off*	*ODM off TCM off*
Effective dose (ICRP103) (mSv)	94.8% (−5.2%)	100.0%	94.8% (−5.2%)	100.0%
Stomach (mGy)	31.5% (−4.7%)	36.2%	31.3% (−4.6%)	35.9%
Colon (mGy)	14.8% (−1.4%)	16.2%	15.0% (−1.4%)	16.4%
Liver (mGy)	10.2% (−0.6%)	10.8%	10.0% (−0.7%)	10.7%
Active bone marrow (mGy)	9.1% (+0.8%)	8.3%	9.3% (+0.9%)	8.4%

**Table 7:** The total effective dose contribution from the given organ for the pelvic region simulated for the large bowtie filter. This is relative to the total effective dose from a procedure where ODM is not enabled with identical ${\text{CTDI}}_{\text{vol}}$ and where TCM is set at the same setting. Only organs which contribute over 5% to the effective dose are shown. The values in brackets indicate the dose change from enabling ODM.

	*Normalized Relative Effective Dose Contribution*			
*Organ*	*ODM on TCM on*	*ODM off TCM on*	*ODM on TCM off*	*ODM off TCM off*
Effective dose (ICRP103) (mSv)	95.4% (−4.6%)	100.0%	95.3% (−4.7%)	100.0%
Colon (mGy)	28.0% (−1.5%)	29.5%	27.7% (−1.5%)	29.2%
Urinary bladder (mGy)	17.9% (−2.9%)	20.8%	18.1% (−3.0%)	21.1%
Ovaries (mGy)	15.4% (−0.2%)	15.6%	15.4% (−0.3%)	15.7%
Active bone marrow (mGy)	17.2% (+1.6%)	15.6%	17.0% (+1.6%)	15.4%
Testicles (mGy)	5.4% (−1.1%)	6.5%	5.5% (−1.1%)	6.6%

**Figure 6:**
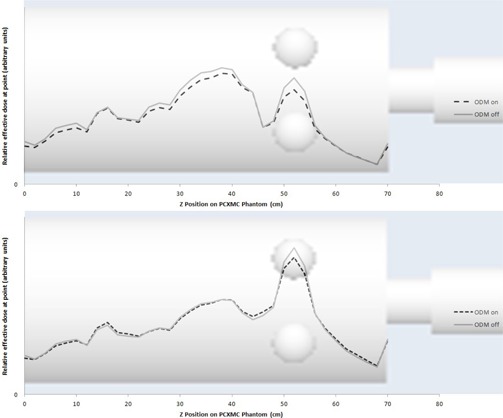
Relative effective dose throughout the PCXMC phantom with (top) and without (bottom) TCM enabled simulated for the large bowtie filter. Results with ODM enabled are increased by a factor of 1/0.8 to give an equivalent ${\text{CTDI}}_{\text{vol}}$ and image quality to exams with ODM disabled.

**Figure 7:**
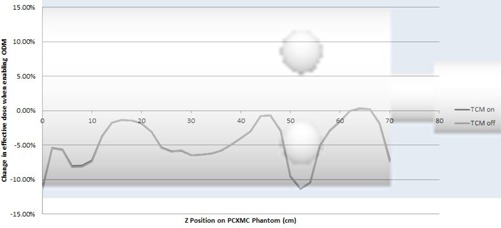
Change in effective dose when ODM is enabled throughout the PCXMC phantom with TCM on and off simulated for the large bowtie filter. Results with ODM enabled are increased by a factor of 1/0.8 to give an equivalent ${\text{CTDI}}_{\text{vol}}$ and image quality to exams with ODM disabled.

## IV. DISCUSSION

ODM was found to reduce the mA in the anterior projections without compensation in the posterior projections, resulting in an increase in image noise (represented by SD) of approximately 14%. Assuming a 0.5 power relationship, based on Poisson detection statistics of image noise with dose, a 20% drop in dose would lead to approximately an 11% increase in noise.

From this analysis it is shown that TCM has a minimal effect on the overall effective dose change from ODM. The lower tube current in the chest region when TCM is enabled has the effect of reducing the dose to the breasts overall, but other radiosensitive organs that also benefit from ODM, such as the stomach, then get a higher proportion of the dose and thus the effective dose is relatively unaffected (when the overall reduction of effective dose from enabling TCM is accounted for).

**Figure 8:**
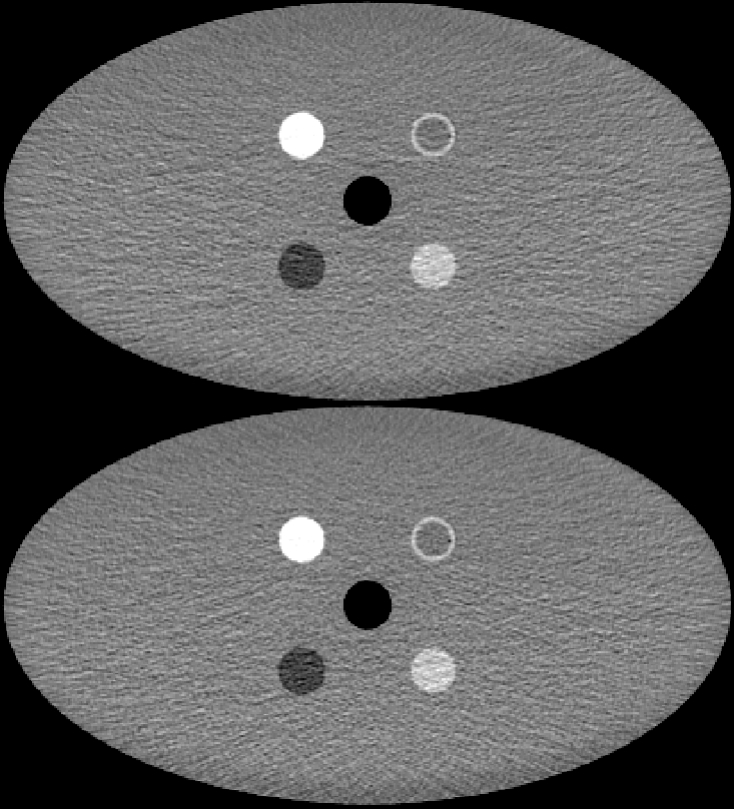
Comparison of the same slice in images of the CelT phantom taken with ODM off (top) and ODM on (bottom) with a CTDI of 1.73 mGy.

Our results indicate that TCM can be used to contribute a small saving in effective dose (approximately 5%), primarily from reductions in equivalent dose to the breasts (reduced by a factor of 0.82), stomach (reduced by a factor of 0.88), the colon (reduced by a factor of 0.93), and the bladder (reduced by a factor of 0.85). In men, the benefit will be smaller (in the region of a 3%–4% reduction in effective dose) as the risk of breast cancer induction is far less. The organ that appears to receive significantly more dose is the active bone marrow (equivalent dose increased by a factor of 1.09). When matching for ${\text{CTDI}}_{\text{vol}}$, no detrimental effects were observed in image quality, both visually (as shown in Fig. 8) and measured using standard deviation and average Hounsfield number. Additionally, no alteration in the texture of the noise was observed by the authors. From these findings, it would be advisable to use ODM on the entirety of the scan region for adult body exams for two reasons. Firstly, effective dose contributions are always lower from the posterior projections. Secondly, enabling ODM for the entire exam will allow matching of ${\text{CTDI}}_{\text{vol}}$ (and therefore noise) to a non‐ODM scan by adjusting the Noise Index, thereby preventing the need to either overdose regions where it is not enabled, or incur a noise penalty in regions where it is enabled.

It is expected that, if this method is introduced clinically, most practitioners will accept the found increase in global image noise. If this is the case, then arguments could be made to support an “across the board” reduction in ${\text{CTDI}}_{\text{vol}}$ for other routine scanning protocols (without the use of ODM) by a simple increase in Noise Index to achieve a 20% dose saving. Unfortunately, ODM is not currently available for either “manual tube current,” or cardiac angiography applications, for which it would be potentially beneficial, as discussed by Loader et al.:^(17)^ “Significant reductions in effective dose for CTCA could therefore be realized by protecting the breast tissue during CT examinations (either by limiting the x‐ray tube exposure time to segments covering the patient's back, utilizing physical breast shields or simply reducing exposure factors)” (p. 248).

Additionally, the inability to use ODM for manual mA scans of the head could limit the ability to reduce the absorbed dose to the lens of the eye for organizations that use fixed mA protocols.

The primary limitation of this study is that it exclusively considered an assortment of phantoms and, therefore, caution should be taken before directly applying the results to patient populations with the associated variation in patient morphology and size. Having said this, the analysis of dose distribution and image quality parameters (such as noise and Hounsfield number) should give a good indication of the functionality of ODM, and the phantom used for patient dose calculations is broadly representative of actual anatomy. It is particularly accurate, for example, in that most radiosensitive organs are located anteriorly. A clinical evaluation of images produced with ODM enabled would ensure that the phantom image quality measurements are replicated in patients.

Further investigations into head and neck regions would yield additional information, particularly as ODM performs differently for head scans (only using a 90° arc). Additionally, it would be beneficial to consider the effect of implementing statistical iterative reconstruction, as this could weigh the projections with lower mA values less significantly.

## V. CONCLUSION

ODM has the effect of a global reduction in ${\text{CTDI}}_{\text{vol}}$ with an associated increase in image noise. The benefit of ODM was found to be reduced when the dose saving contribution from the reduced ${\text{CTDI}}_{\text{vol}}$ was removed. Given that there is a higher contribution to effective dose throughout the body from the anterior projections, consideration should be given to applying ODM throughout the scan volume if it is used. However, given that the overall benefit is small to both individual organs and the effective dose, departments may consider focusing optimization elsewhere.

## COPYRIGHT

This work is licensed under a Creative Commons Attribution 4.0 International License.

## Supporting information

Supplementary Material

Supplementary Material

Supplementary Material
