# Supplementary figures and images for: An evaluation of organ dose modulation on a GE optima CT660‐computed tomography scanner
Source: J Appl Clin Med Phys. 2016 May 8;17(3):380–91. doi: 10.1120/jacmp.v17i3.5724 (PMC5690941; doi:10.1120/jacmp.v17i3.5724)

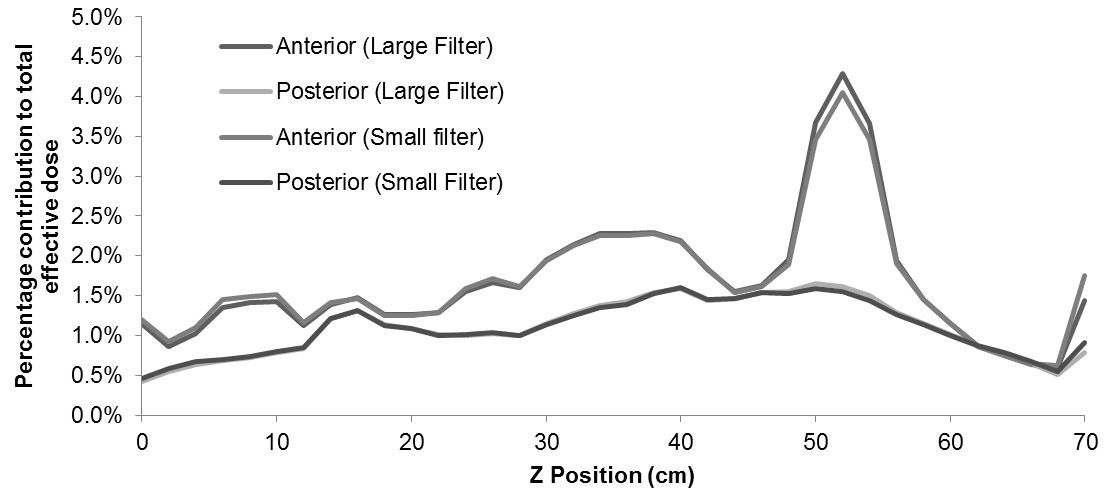

Supplement: Supplementary file 1 — Supplementary Material [file ACM2-17-380-s001.png]

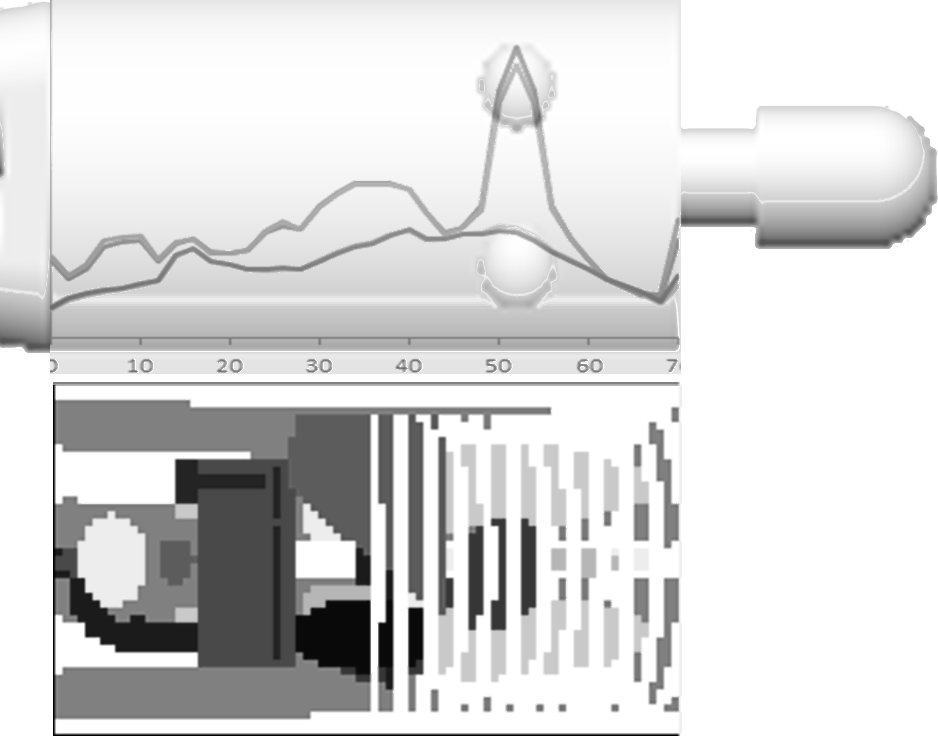

Supplement: Supplementary file 2 — Supplementary Material [file ACM2-17-380-s002.png]

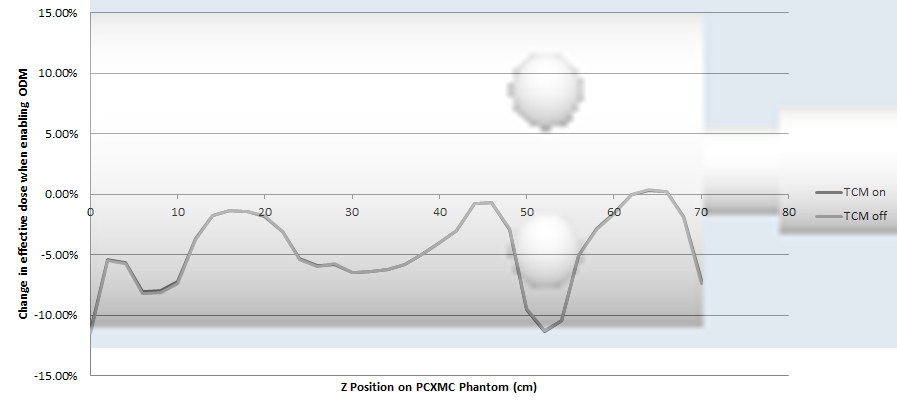

Supplement: Supplementary file 3 — Supplementary Material [file ACM2-17-380-s003.png]
